# Antibiotic-Resistant Bacteria in Hydroponic Lettuce in Retail: A Comparative Survey

**DOI:** 10.3390/foods9091327

**Published:** 2020-09-21

**Authors:** Kit-Ling Lam, Wai-Po Kong, Po-Yi Ling, Tsz-Ho Lau, Kin-Hang Ho, Fred Wang-Fat Lee, Ping-Lung Chan

**Affiliations:** Department of Science, School of Science and Technology, The Open University of Hong Kong, Ho Man Tin, Hong Kong; s1097651@ouhk.edu.hk (K.-L.L.); wai-po-ball.kong@connect.polyu.hk (W.-P.K.); s1204354@ouhk.edu.hk (P.-Y.L.); thlau@ouhk.edu.hk (T.-H.L.); s1223653@ouhk.edu.hk (K.-H.H.); wflee@ouhk.edu.hk (F.W.-F.L.)

**Keywords:** hydroponic produce, organic produce, lettuce, antibiotic-resistant bacteria, antibiotic resistance gene, urban agriculture, sustainability, tetracycline, sulphadiazine

## Abstract

Hydroponic produce is gaining popularity due to its suitability for urban agriculture. The general public also considers that hydroponic produce is free from microbiological contamination. In this study, we compared the frequency and abundance of tetracycline-resistant and sulphadiazine-resistant bacteria and the minimal inhibitory concentration (MIC) of these isolates in conventional, organic, and hydroponic lettuce sold in retail. We also determined the frequency of samples carrying *tetB*, *tetX*, *sul1*, *sul2*, and *int1* genes by PCR and further quantified the copy number of *tetX*, *sul1*, and *int1* genes in samples positive for these genes using qPCR. As expected, the number of resistant bacteria and the MICs of these isolates were lowest in hydroponic lettuce and highest in organic lettuce. All tested resistant genes, except *int1*, were detected in samples of all three production methods, but no significant difference was observed between the three groups in the frequency of samples carrying the resistance genes examined or in their copy number. To the best of our knowledge, it is the first study directly reporting the existence of antibiotic-resistant bacteria and resistance genes in hydroponic vegetables sold in retail. The result highlights that the risk of antibiotic-resistant bacteria contamination in hydroponic produce should be further investigated.

## 1. Introduction

Hydroponic vegetables are grown in soilless systems and rely on the use of nutrient solutions to support their growth. This form of agriculture is particularly suitable for the vertical agriculture system used in industrialised and local urban farming systems [1,2,3] because of its efficiency in land and water usage [4] and increased yield and economic benefits [5]. Barbosa et al. compared the yields and water requirement of lettuce grown hydroponically and conventionally and their data revealed that hydroponic production increased the yield by 11-fold and reduced water usage by 12.5-fold [5]. Apart from increased production yield and efficiency, hydroponic agriculture can also be integrated as part of wastewater treatment measures. Jesse et al. investigated the potential of using treated post-hydrothermal liquefaction wastewater for producing hydroponic lettuce and demonstrated that wastewater filtered with sand and supplemented with hydroponic fertiliser supported the growth [6]. A similar effect was also observed in lettuce grown with wastewater supplemented with mineral fertiliser [7]. Due to these unique features, it is argued that the hydroponic agriculture system is more sustainable than conventional agriculture system and is one of the means to strengthen urban food security and city resilience against the impact brought by climate change [8,9,10]. Hydroponic produce is not only gaining popularity among farmers and policymakers but is also increasingly favoured by customers. The increased popularity is primarily based on the belief that hydroponic produce is more hygienic and is free from microbiological contamination since, as a form of industrialised agriculture system, the environment and materials used are tightly controlled [2].

This belief was examined in several studies, and conflicting results were obtained. In a three-month study, Riser et al. examined the bacterial load in hydroponic lettuce, nutrient solution, and peat-vermiculite growing mixture. No typical foodborne pathogen was detected in that study, and it was concluded that hydroponic lettuce posed no microbiological hazard [11]. However, Wang et al. isolated Shiga toxin-producing *E. coli* from the leaf surface of hydroponic lettuce [12]. Dankwa also isolated 4.1 log CFU per g of aerobic bacteria by aerobic plate count from the leaves of harvested hydroponic lettuce [13]. These findings suggested that microbiological contamination of hydroponic produce is possible. Apart from microbiological contamination, hydroponic produce may also be contaminated by xenobiotics in nutrient solution. Although, to the best of our knowledge, there is no direct investigation on the existence of antibiotics in hydroponic nutrient solutions, several hydroponic experiments indicated that antibiotics added to nutrient solution were absorbed and accumulated in plants [14,15,16]. Zhang et al. also demonstrated in a hydroponic experiment that addition of antibiotics in nutrient solution induced antibiotics resistance and increased the abundance of various resistance genes in the endophytic system of pak choi grown hydroponically [17].

The findings from the above studies lead to a hypothesis that hydroponic vegetables in retail may harbour antibiotic-resistant bacteria. Given that tetracycline and sulphonamide are the most used veterinary antibiotics and are also present in the environment in high concentrations [18], in this study, we aimed to compare the frequency and abundance of tetracycline-resistant and sulphadiazine-resistant bacteria and the minimum inhibitory concentration of these bacteria from conventional, organic, and hydroponic lettuce sold in retail in Hong Kong. The frequency of samples positive for *tetB*, *tetX*, *sul1*, *sul2*, and *int1* and the copy number of *tetX*, *sul1*, and *int1* genes between the lettuce of the three production methods were also determined.

## 2. Materials and Methods

### 2.1. Sampling

Pre-packaged conventional, organic and hydroponic romaine lettuce (*Lactuca sativa*) were purchased from local supermarkets in Hong Kong. Ten lettuce samples of each production method (thirty samples in total) were purchased for the isolation and enumeration of antibiotic-resistant bacteria and the determination of the minimum inhibitory concentration of these isolates. Another twenty lettuce samples of each production method (total sixty samples) were purchased for the detection and quantification of antibiotic resistance genes. See Table S1 for detailed information of each sample.

### 2.2. Isolation and Enumeration of Tetracycline-Resistant and Sulphadiazine-Resistant Bacteria

The isolation and enumeration of tetracycline-resistant and sulphadiazine-resistant bacteria in lettuce samples was performed using a method modified from methods described by Marti et al. and Esiobu et al. [19,20]. Briefly, lettuce leaves were cut into small pieces using autoclaved scissors. Fifty grams of the samples were homogenised in a sterile stomacher bag with 100 mL autoclaved water at 200 rpm for 2 min. The homogenates were diluted with autoclaved deionised water by tenfold. The undiluted and diluted homogenate were added to Mueller–Hinton agar (Sigma-Aldrich, St. Louis, MO, USA) containing 4 µg/mL tetracycline (Sigma-Aldrich, St. Louis, MO, USA) and Mueller–Hinton agar containing 1024 µg/mL sulphadiazine (Sigma-Aldrich, St. Louis, MO, USA) in triplicate and incubated at 37 °C for 48 h.

### 2.3. Determination of Minimal Inhibitory Concentration of Resistant Bacteria Isolates

The minimum inhibitory concentration (MIC) of tetracycline-resistant and sulphadiazine-resistant bacteria isolated above was determined by the standard broth microdilution method (Clinical and Laboratory Standards Institute Standard M07) [21]. For each sample with tetracycline-resistant bacteria or sulphadiazine-resistant bacteria isolated in agar plate, four colonies of tetracycline-resistant or sulphadiazine-resistant bacteria were selected randomly. The MIC of these four colonies were determined as follows: each colony was suspended in 200 µL phosphate-buffered saline (Sigma-Aldrich, St. Louis, MO, USA) and adjusted to 0.5 McFarland standard. The bacterial suspension contained about 1 × 10^8^ CFU/mL was diluted to 1 × 10^6^ CFU/mL with Mueller–Hinton broth (Sigma-Aldrich, St. Louis, MO, USA). Fifty microliters of the bacterial suspension were pipetted into each well of a 96-well plate (Corning, Corning, NY, USA). In each well of the 96-well plate contained fifty microliters of tetracycline solution (8–1024 g/mL) and sulphadiazine solution (2048–65,536 g/mL). The inoculum was incubated at 37 °C for 18 h, and the absorbance at 600 nm was measured. The MIC value of the four colonies were averaged and was used represent the MIC value of that sample. Three technical replicates were performed for each colony tested.

### 2.4. DNA Extraction

The method of DNA extraction was modified from the method described by Marti et al. [19]. Briefly, lettuce leaves were cut into small pieces using autoclaved scissors. Fifty grams of the samples were homogenised in a sterile stomacher bag with 100 mL autoclaved water at 200 rpm for 2 min. Forty millilitres of the homogenate was centrifuged at 4,000× *g* for 5 min. DNA was extracted from the resulting pellet using the DNeasy PowerSoil Pro kit (Qiagen, Germantown, MA, USA) according to the manufacturer’s instructions.

### 2.5. Detection of Antibiotic Resistance Genes

All PCR assays were conducted in a 25-µL volume reaction using a C1000 Touch thermal cycler (Bio-rad, Hercules, CA, USA). The PCR mixture consisted of 2.5 µL of 10X Taq buffer (Thermo-Scientific, Waltham, MA, USA), 1.5 µL of MgCl_2_ (25 mM), 1µL of dNTP mix (10 mM), 0.7 µL of each primer (10 µM), 0.25 µL of Taq polymerase (recombinant) (5 U/µL), 1 µL of DNA template (100 ng/µL), and nuclease-free water for the remaining volume. DNA denaturation at 95 °C for 3 min was followed by 35 amplification cycles (94 °C (40 s), 56 °C for *tetB*, *sul2*, and *int1*/60 °C for *tetX* and *sul1* (30 s), and 72 °C (1 min)), and a final extension step at 72 °C for 10 min. PCR detection of antibiotic resistance genes was duplicated for each sample. Samples were considered positive for a resistance gene if the gene was detected once. See Table S2 for the primer sequences and Table S3 for the sequencing results of the amplified PCR products.

### 2.6. Quantitation of tetX, sul1, and int1

Resistance genes were quantified in triplicate using the StepOne Real-Time PCR System (Applied Biosystems, Waltham, MA, USA) and fluorescent dye SsoAdvanced Universal SYBR-Green Supermix (Bio-rad, Hercules, CA, USA). Each qPCR reaction was performed in a 10 µL volume containing 5 µL of SYBR Supermix (Bio-rad, Hercules, CA, USA), 0.3 µL of each primer (10 µM), 0.5 µL of DNA template (100 ng/µL) for *tetX* and *sul1* or 1 µL for *int1*, and nuclease-free water for the remaining volume. DNA denaturation at 95 °C for 15 min was followed by 40 amplification cycles (95 °C (15s), 67 °C, 67.5 °C, and 64.7 °C for *tetX*, *sul1*, and *int1*, respectively (35 s), and 72 °C (30 s)). See Table S2 for the primer sequences and Table S3 for the sequencing results of the amplified qPCR products.

### 2.7. Statistical Analysis

Graphs and statistical analyses were performed using Prism 8.0 for Mac software (GraphPad Software, San Diego, CA, USA). Chi-square test was used for comparing the frequency of lettuce samples positive for resistant bacteria and the frequency of lettuce samples positive for each resistance gene. One-way ANOVA with a Kruskal–Wallis comparison test were used for comparing the number of resistant bacteria, the minimum inhibitory concentration of the resistant-isolates, and the copy number of *tetX* and *sul1* between the lettuce of the three production methods. Two-way ANOVA with Sidak’s comparison test were used for comparing the difference in the copy number of *tetX* and *sul1* between lettuce of the three production methods. A Mann–Whitney test was used for comparing the copy number of *int1* between conventional and organic lettuce samples. A *p*-value of <0.05 was considered to be significant.

## 3. Results

### 3.1. Frequency and Number of Antibiotic-Resistant Bacteria in Lettuce Samples

**Figure 1:**
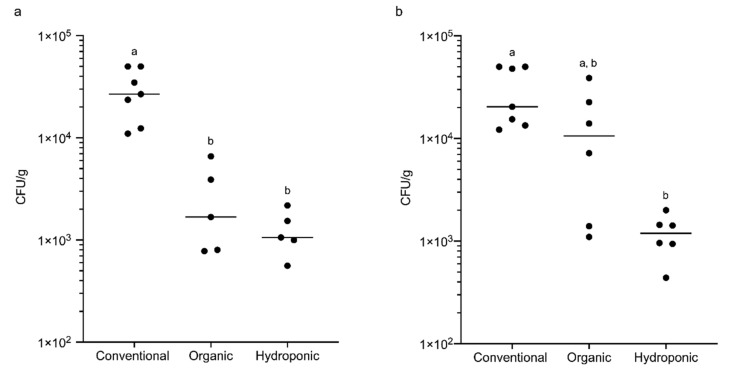
The number of antibiotic-resistant bacteria isolated from lettuce of three different production methods: (**a**) tetracycline-resistant bacteria; (**b**) sulphadiazine-resistant bacteria (ten samples for each production method). Different letters indicate a significant difference (*p* < 0.05), one-way ANOVA with a Kruskal–Wallis comparison test.

We first quantified and compared the frequency and number of tetracycline-resistant and sulphadiazine-resistant bacteria between lettuce of the three production methods. No significant difference in the frequency of samples harbouring tetracycline-resistant and sulphadiazine-resistant bacteria was observed between the lettuce of the three production methods (Table 1). However, more tetracycline-resistant bacteria were found in conventional lettuce (2.980 × 10^4^ CFU/g) than in organic (2.752 × 10^3^ CFU/g; *p* < 0.05) and hydroponic (1.268 × 10^3^ CFU/g; *p* < 0.01) lettuce (Figure 1a). Conventional lettuce also harboured more sulphadiazine-resistant bacteria (2.999 × 10^4^ CFU/g) than hydroponic lettuce (1.200 × 10^3^ CFU/g; *p* < 0.01) but no significant difference was found between conventional lettuce and organic lettuce in the number of sulphadiazine-resistant bacteria (1.418 × 10^4^ CFU/g) (Figure 1b).

### 3.2. Minimum Inhibitory Concentration of Resistant Isolates

**Figure 2:**
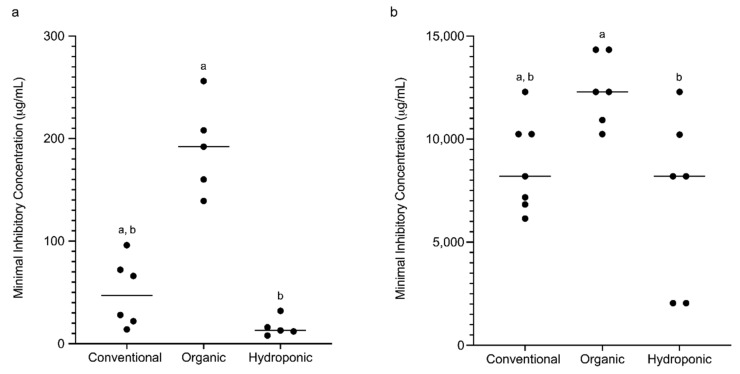
Minimum inhibitory concentration (MIC) of resistant bacteria isolated from lettuce samples: (**a**) tetracycline; (**b**) sulphadiazine. Different letters indicate a significant difference (*p* < 0.05), one-way ANOVA with a Kruskal–Wallis comparison test.

The minimum inhibitory concentration (MIC) of the isolated resistant bacteria was also determined. Four colonies were randomly picked from each sample positive for resistant bacteria, and the tetracycline or sulphadiazine MIC of these bacteria was determined. Interestingly, although conventional lettuce had the highest number of tetracycline-resistant and sulphadiazine-resistant bacteria, our result indicated that the isolates from organic lettuce had the highest MIC for both antibiotics, while the MIC of hydroponic lettuce for the two antibiotics was the lowest. The mean tetracycline MIC of isolates from organic lettuce was 191.0 µg/mL and was significantly higher than that of hydroponic lettuce (16.2 µg/mL; *p* < 0.01), but no significant difference was observed between organic and conventional lettuce (49.7 µg/mL) (Figure 2a). A similar phenomenon was also observed in sulphadiazine-resistant bacteria. The mean sulphadiazine MIC of isolates from organic lettuce was 12,402 µg/mL and was significantly higher than that of isolates from hydroponic lettuce (7,163 µg/mL; *p* < 0.05). Similarly, no significant difference was obtained between organic and conventional lettuce (8,728 µg/mL) (Figure 2b).

### 3.3. Frequency of Lettuce Samples Carrying Antibiotic Resistance Genes

We further examined the frequency of lettuce carrying different resistance genes. Twenty lettuce samples of each production method were purchased from local supermarkets. We compared the frequency of samples carrying *tetB*, *tetX*, *sul1*, *sul2*, and *int1* in the three groups by PCR. No significant difference was found in all genes tested. *tetB* was found in 13 conventional lettuce samples and 10 of both organic and hydroponic lettuce samples (Table 2). *tetX* was found in 16, 14, and 13 samples of conventional, organic, and hydroponic lettuce, respectively. For *sul1*, 17, 18, and 19 samples of conventional, organic, and hydroponic lettuce were found to be carrying the gene, respectively, while 17, 16, and 19 samples of conventional, organic, and hydroponic lettuce were found to be carrying *sul2*, respectively. However, *int1* was only found in conventional and organic lettuce samples (6 and 3 samples, respectively).

### 3.4. Copy Number of Antibiotic Resistance Genes

**Figure 3:**
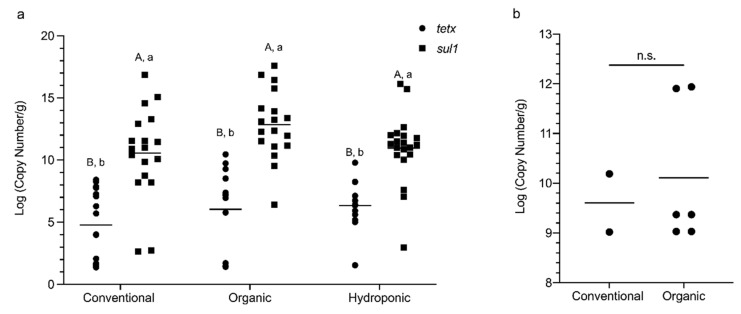
Copy numbers of *tetX*, *sul1*, and *int1* in lettuce samples: (**a**) comparison of the copy number of *tetX* and *sul1* between lettuce of the three production methods by two-way ANOVA with Sidak’s test. Uppercase letters denote comparison between lettuce of the three production methods and lowercase letters denote comparison between the two resistance genes. Different letters indicate a significant difference (*p* < 0.05); (**b**) comparison of the copy number of *int1* between the conventional and organic lettuce by Mann–Whitney test. No significant difference was found.

The copy numbers of *tetX*, *sul1*, and *int1* in the lettuce of the three production methods were also determined by qPCR and were compared between the three groups, except the *int1* in hydroponic lettuce, which was omitted, since *int1* was not detected in this group. Although no significant difference was found between lettuce of the three production methods in the copy number of *tetX* and *sul1*, the copy number of *sul1* was consistently higher than that of *tetX* in all three groups. The log of the copy number of *tetX* and *sul1* in conventional lettuce was 4.772 copies/g and 10.500 copies/g, respectively (*p* < 0.001). In organic lettuce, the log of the copy number of *tetX* and *sul1* was 6.040 copies/g and 12.605 copies/g, respectively (*p* < 0.001). In hydroponic lettuce, the log of the copy number of *tetX* and *sul1* were 6.346 copies/g and 10.680 copies/g, respectively (*p* < 0.04) (Figure 3a). A similar phenomenon was not observed for *int1*, which the log of the copy numbers in conventional and organic samples were 9.119 copies/g and 9.500 copies/g, respectively, and no significant difference was detected (Figure 3b).

## 4. Discussion

In this study, we aim to shed light on antibiotic-resistant bacteria contamination in hydroponic produce in retail and to juxtapose it with that of conventional and organic lettuce in retail.

We first surveyed the frequency and number of bacteria resistant to tetracycline and sulphadiazine in the lettuce of the three production methods. About 50–70% of samples harboured resistant bacteria in the lettuce of the three production methods and no significant difference was found between them. The high frequency of samples harbouring resistant bacteria indicated that contamination of lettuce by resistant bacteria is common, irrespective of cultivation methods. It was observed that hydroponic lettuce samples harboured the least number of resistant bacteria among the three groups. This result is not surprising as hydroponic produce is grown in a soilless system, and, generally, the environment and the material used are controlled and defined. These features may contribute to the result in two ways. First, the use of soilless controlled environment and defined materials may generate a more hygienic environment, leading to a lower bacterial load in the environment and thus, in turn, a lower number of bacteria on the produce. Manzocco et al. illustrated that the number of coliforms, *pseudomonas*, and *Enterobacteriaceae* bacteria in hydroponic lettuce was about 18–55% lower than that of lettuce cultivated in a soil system [22]. Moreover, since the water and the nutrient solution used are supposed to be free of antibiotics, antibiotic-resistant bacteria are unlikely to be selected in the system and contaminating the environment and the produce. Surprisingly, conventional lettuce in this study harboured the highest number of tetracycline-resistant and sulphadiazine-resistant bacteria. It was expected that organic lettuce harboured the highest number of antibiotic-resistant bacteria due to the use of manure and organic fertilisers [23]. The higher number of tetracycline-resistant and sulphadiazine-resistant bacteria in conventional lettuce samples may reflect a lower concentration of tetracycline and sulphadiazine in these samples compared with that of the samples of organic lettuce. A lower concentration of antibiotics on lettuce surface may exert a lower selective pressure on the bacteria on the lettuce surface. This may have allowed bacteria with lower resistance to survive and then end up being isolated in our experiment. In contrast, a higher concentration of antibiotics on lettuce surface may exert a strong selective pressure on the bacteria on the lettuce surface. This may render only bacteria with higher resistance to survive, and thus a lower number of resistant bacteria can be isolated.

In fact, our data indicated that the average MIC of the resistant bacteria isolated from conventional lettuce for the two antibiotics was lower than that of the resistant bacteria isolated from organic lettuce, although no statistical significance was obtained. Our data also indicated that the MIC of the resistant bacteria isolated from hydroponic lettuce for the two antibiotics was the lowest. As discussed in the above, the MIC result is likely related to the concentration of tetracycline and sulphadiazine on the lettuce surface, which selects bacteria with high MIC [24] and suggests that organic lettuce samples were exposed to a higher concentration of tetracycline and sulphadiazine while hydroponic lettuce samples were exposed to a lower concentration. The high concentration of tetracycline and sulphadiazine in organic lettuce may originate from the manure and organic fertilisers applied as these materials were derived from animals, which may be fed with feeds containing antibiotics. On the other hand, although the number of resistant bacteria and the MIC of these isolates were the lowest in hydroponic lettuce, the mere existence of antibiotic-resistant bacteria in hydroponic lettuce is alarming, particularly when the mean tetracycline MIC of the bacteria isolated from the hydroponic lettuce was up to 16.2 µg/mL, which is higher than the resistant breakpoint for *Salmonella* and *Escherichia coli* [25]. The sulphadiazine MIC of bacteria isolated from hydroponic lettuce is also higher than the resistant breakpoint [26]. Tetracycline-resistant and sulphadiazine-resistant bacteria were isolated from nutrient solutions (data not shown), suggesting that nutrient solution may be a possible source of the isolated resistant bacteria.

We further compared the frequency of samples positive for *tetB*, *tetX*, *sul1*, *sul2*, and *int1*, and the copy number of *tetX*, *sul1*, and *int1* as it was reported that the copy number of resistance genes correlates with antibiotics concentration in drinking water and hydroponic vegetables [17,27]. A meta-analysis also suggested that antibiotic selection pressure, as a function of antibiotic concentration, is positively correlated to the abundance of antibiotic resistance gene [28]. The copy number of *sul1* was consistently higher than that of *tetX* in all three groups, suggesting that all the produce may be exposed to a higher concentration of antibiotics of sulphonamide class. No significant difference was found in the copy number of the two resistant genes across the three groups. This suggests that the antibiotic concentration in the lettuce of the three groups were similar and contradicts the hypothesis that the antibiotic concentration of the three groups of lettuce were different. It was reported that it is the expression level of resistance genes, instead of the abundance, correlated with the antibiotic concentration at low concentration levels [29]. It is thus possible that the abundance of antibiotic-resistance genes only correlates with antibiotic concentrations at high concentration levels. It was also reported that the copy number of resistance genes was not correlated with the concentration of antibiotics [30], and may be the result of the higher stability of sulphonamide resistance genes compared with that of tetracycline resistance genes [31]. Taken together, contaminating antibiotics from environment or raw materials may contribute to the selection of resistant bacteria with high MICs and other environmental factors may contribute the stability of resistance genes. Environmental factors may thus be the critical factors affecting the resistance of bacteria.

To the best of our knowledge, this is the first direct report of the detection of antibiotic-resistant bacteria and resistance genes in commercially available hydroponic produce. Although the sample size of this study was small and a larger survey with a higher statistical power will be needed, our data indicated that antibiotic-resistant bacteria contamination is common in hydroponic produce and the threat cannot be ignored. Control measures, particularly the control of the production environment and the production of nutrient solution and other materials, must be put in place. To reduce bacterial contamination, the water should not be reused, at least not before disinfection. Ehret et al. pointed out that the disinfection of recirculating nutrient solution can reduce the bacterial load in nutrient solution [32]. Water taps were shown to be a source of microbiological contamination and thus should be disinfected regularly [33]. Marques proposed the use of internet of things (IoT) for the real-time monitoring and control of different environmental parameters on a hydroponic farm [34]. The seed surface may also be another source of bacterial contamination. Pre-treatment of the seed surface may effectively reduce the risk of microbial contamination [35]. In long term, a more comprehensive survey and surveillance covering more antibiotics and resistance genes is warranted to determine and monitor the extent of antibiotic contamination in hydroponic vegetables. A transition from hydroponic agriculture to controlled environment agriculture and the development of relevant standards and good practice guidelines will also be crucial for mitigating the risk of bacterial contamination and to safeguard the safety of customers and retain their confidence in hydroponic produce.

## Figures and Tables

**Table 1:** Frequency of lettuce samples harbouring resistant bacteria ^1^.

	Tetracycline-Resistant	Sulphadiazine-Resistant
Conventional	7	7
Organic	5	6
Hydroponic	5	6

^1^ Ten samples for each production method.

**Table 2:** Frequency of lettuce samples carrying resistant genes ^1^.

	*tetB*	*tetX*	*sul1*	*sul2*	*int1*
Conventional	13	16	17	17	6
Organic	10	14	18	16	3
Hydroponic	10	13	19	19	0

^1^ Twenty samples for each production method.

## Supplementary Materials

The following are available online at https://www.mdpi.com/2304-8158/9/9/1327/s1, Table S1: Detailed information of purchased lettuce samples, Table S2: Sequences of primers used. Table S3: Sequencing results of amplified resistance genes.
